# Countrywide climate features during recorded climate-related disasters

**DOI:** 10.1007/s10584-019-02556-w

**Published:** 2019-12-04

**Authors:** Elisabeth Tschumi, Jakob Zscheischler

**Affiliations:** 1 Climate and Environmental Physics and Oeschger Centre for Climate Change Research, University of Bern, Bern, Switzerland; 2 Institute for Atmospheric and Climate Science, ETH Zurich, Zurich, Switzerland

**Keywords:** Disaster, Climate extreme, Drought, Flood, Heat wave, Cold wave

## Abstract

Climate-related disasters cause substantial disruptions to human societies. With climate change, many extreme weather and climate events are expected to become more severe and more frequent. The International Disaster Database (EM-DAT) records climate-related disasters associated with observed impacts such as affected people and economic damage on a country basis. Although disasters are classified into different meteorological categories, they are usually not linked to observed climate anomalies. Here, we investigate countrywide climate features associated with disasters that have occurred between 1950 and 2015 and have been classified as droughts, floods, heat waves, and cold waves using superposed epoch analysis. We find that disasters classified as heat waves are associated with significant countrywide increases in annual mean temperature of on average 0.13 ^∘^C and a significant decrease in annual precipitation of 3.2%. Drought disasters show positive temperature anomalies of 0.08 ^∘^C and a 4.8 % precipitation decrease. Disasters classified as droughts and heat waves are thus associated with significant annual countrywide anomalies in both temperature and precipitation. During years of flood disasters, precipitation is increased by 2.8 %. Cold wave disasters show no significant signal for either temperature or precipitation. We further find that climate anomalies tend to be larger in smaller countries, an expected behavior when computing countrywide averages. In addition, our results suggest that extreme weather disasters in developed countries are typically associated with larger climate anomalies compared to developing countries. This effect could be due to different levels of vulnerability, as a climate anomaly needs to be larger in a developed country to cause a societal disruption. Our analysis provides a first link between recorded climate-related disasters and observed climate data, which is an important step towards linking climate and impact communities and ultimately better constraining future disaster risk.

## Introduction

Disasters lead to thousands of lost lives every year and billions of dollar in damages (Guha-Sapir et al. 2017; Handmer et al. 2012). Climate-related disasters associated with extreme weather events such as droughts, floods, heat waves, and cold waves are responsible for a large part of these impacts (Guha-Sapir et al. 2017). Climate-related disasters typically occur as the response to a climate hazard, the exposure of goods and people to this hazard, and the specific vulnerability of exposed people, infrastructure, and environment (Oppenheimer et al. 2014). The climate science community is continuously providing a better understanding of climate extremes and climate-related hazards. Readily available climate data from observations and model projections allow for a comparably easy assessment of climatic hazards (IPCC 2012, 2014). In contrast, the disaster risk community argues that vulnerability, for instance related to culture, social systems, or governance, is at least as important as the hazard component for determining disaster risk, albeit much less understood and quantified (Blaikie et al. 2004; Peduzzi 2019). Given these different assessments, there is still a wide gap between the information climate scientists provide and its application and usefulness for efforts aiming at disaster risk reduction.

With ongoing climate change and the continuing emission of greenhouse gases, many extreme weather events have and will become more frequent and more intense (Seneviratne et al. 2012). However, not only climate hazards are changing. Exposure changes concurrently because of population growth and urbanization (Jones et al. 2015; Smirnov et al. 2016). Climate change ultimately also alters vulnerability by affecting livelihoods (Morton 2007), food security, and health of communities around the world (Oppenheimer et al. 2014). In developing countries, more people die due to climate-related disasters (Kahn 2005). Developing countries are generally less able to adapt to climate change and sectors like human health or food security are more exposed to variability in the climate system (Füssel 2010).

Well-informed disaster risk assessments and projections require knowledge on how climate anomalies are linked to disasters, and which regions are most vulnerable to which type of climate hazard. Currently, no general description of these relationships exist for past disasters. Although many studies are available for individual events with detailed descriptions of the driving weather conditions (e.g., Barriopedro et al. 2011; Hoyos et al. 2013; Cramer et al. 2014), it is very difficult to derive more general relationships that hold for larger areas and different types of disasters from these case studies. Deriving robust statistics to link disasters with their dominant climatic drivers are only possible with large samples consisting of multiple events. Attributing climate impacts to climate anomalies is challenging (Zscheischler et al. 2013) and a quantitative attribution of the occurrence of disasters to climatic anomalies is currently lacking. Only recently, disasters associated with droughts and extreme heat have been linked to substantial crop reductions (Lesk et al. 2016). While these results provide some insights on the possible pathways of climatic hazards toward the occurrence of a disaster, no climate information was used in the analysis. This is because disaster databases usually do not record climate information but only impacts, which are then associated with weather events via news reports rather than a statistical analysis.

**Fig. 1:**
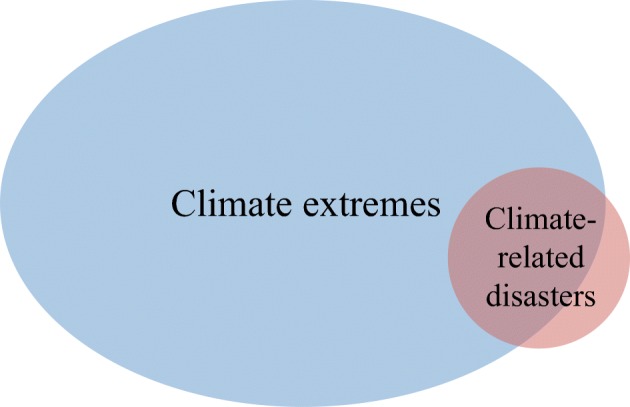
Climate extremes, as typically defined by the climate community, denote a large number of event types that are defined based on climate variables at various temporal scales. Climate-related disasters may be caused by a climate extreme. However, overall, only a very small fraction of climate extremes leads to a disaster. Furthermore, climate-related disasters can be caused by climate anomalies that are not captured by traditional climate extreme analyses, for instance when vulnerability is very high such that even a small climate anomaly can lead to a large impact

The Emergency Events Database (EM-DAT) records disasters and their impacts back to the year 1900 (Guha-Sapir et al. 2017). Within the disasters classified as “natural,” further subcategories exist that associate disasters with droughts, floods, heat waves, and cold waves. The information on disaster impacts and their association to climatic causes is collected from UN agencies, non-governmental organizations, and insurance companies, besides others. The exact source of information for each disaster is not available, however. This means that reports may be biased and the information, especially impact numbers, should be treated with caution. For instance, depending on where the information about a disaster comes from, there might be an interest to increase or decrease associated impacts depending on the political situation, and some disaster might not be reported at all (Guha-Sapir and Checchi 2018). To our knowledge, no comprehensive analysis about the limitations and uncertainties associated with EM-DAT is available at this point. However, since only date and location are used in this study, the uncertainties associated with impact numbers are not of concern. Because the classification of disasters is not based on information on climatological phenomena derived from climate observations, it is unclear to which extent disasters are associated with actual climate anomalies. Furthermore, disasters per definition only occur if a measurable impact happens. Hence, even if they are caused by a climate extreme, they only sample a very small and potentially highly unrepresentative fraction of the actual set of climate extremes. Some climate-related disasters may even be related to climatic events that would not be considered extreme in a meteorological sense, as moderate climate anomalies can lead to large impacts in situations where vulnerability is exceptionally high due to political or other reasons (Tauger 1991; Gasparrini et al. 2015; Delbiso et al. 2017; Scovronick et al. 2018) or when multiple drivers come together (Leonard et al. 2014). For instance, Delbiso et al. (2017) found that minimal food insecure areas showed higher under five death rates compared to stressed food insecure areas in Ethiopia. Similarly, Gasparrini et al. (2015) found in a multicountry observational study that most deaths are caused by exposure to moderately hot and cold temperatures, whereas the contribution of extreme days is comparatively low. These findings may indicate that for severe climate hazards, communities have protection mechanisms in place or rely on international help, in contrast to more moderate hazards. Figure 1 provides a conceptual illustration of the overlap between climate extremes and climate-related disasters. Disasters are very rare in general, and their occurrence is highly dependent on the local environmental conditions. Yet, precise information on the location and timing of a disaster is often not available in commonly used disaster databases such as EM-DAT. Because of lagged effects—a drought might cause crop failure that leads to starvation half a year later—assigning a time period to a disaster is not straightforward. For all of these reasons, it is very difficult to derive general conclusions about the relationships between climate-related disasters and their contributing weather and climate events.

Despite the challenges, establishing a statistical relationship between the occurrence of disasters and climate anomalies is an important step for improving future climate risk assessments. First, one may be able to separate the contribution of the climatic hazard in relation to vulnerability and exposure to the overall risk. This may help to identify regions where climatic hazards are particularly important. Second, such an analysis may pave the way to more informative indicators of climate risks (Sillmann et al. 2018). Third, associating disasters with climate events may identify important compounding effects in the climate system (Zscheischler et al. 2018). To make a first step towards such an analysis, here we investigate whether past climate-related disasters as recorded by EM-DAT can be associated with anomalies in temperature and precipitation. The limited amount of information on the exact location and timing of the recorded disasters in EM-DAT only allow an analysis at country basis and yearly time scale. Given the extensive averaging to country scale, we expect an influence of the country size on the magnitude of the climate anomaly. Furthermore, the wealth of a country can be used as an indicator for its vulnerability to climate anomalies. We thus expect that in poorer countries smaller climate anomalies suffice to cause disasters, as their ability to cope with climate variability is limited.

## Data and methods

We use information on the country and year of recorded disasters from EM-DAT, which is maintained by the Centre for Research on the Epidemiology of Disasters (CRED) in Brussels, Belgium. EM-DAT is an open-access database and records disasters worldwide on a country-level base. To be classified as a disaster, an event needs to fulfill at least one of the following four conditions: (i) 10 or more people dead, (ii) 100 or more people affected, (iii) a declaration of a state of emergency, or (iv) a call for international assistance. These criteria may be different for other disaster databases as there is no single accepted definition of a disaster.

The recorded information includes country and year of the disaster, which is what was used for this study, as well as other variables such as damage in dollars and number of people affected/killed. We expect all this information to be subject to some uncertainty, but we assume the date and location of the disaster to be reliable. We focus on disasters that are classified as being related to precipitation and temperature extremes, namely droughts, floods, heat waves, and cold waves. Within EM-DAT, droughts are “Climatological” and floods are “Hydrological” events, while extreme temperature events are classified as “Meteorological.” The public version of EM-DAT generally only records the country and year in which the disaster took place. For some events, it also records a sub-region or a month, but not for all. More detailed information on the location is missing for 28 % of the disasters classified as droughts, 8 % of the disasters classified as floods, 41 % of the disasters classified as heat waves, and 35 % of the disasters classified as cold waves. Detailed information on the start and ending time is available for slightly more than the above cases. In order to not restrict our sample size, we perform the analysis at a country basis with a yearly time scale. For more detailed information on EM-DAT, see www.emdat.be.

For brevity, we sometimes refer to the examined disasters as droughts, floods, heat waves, and cold waves according to their EM-DAT classification. However, note that there is actually no established link between an EM-DAT event classified as “drought” and a climatological drought. This also holds for the other disaster types. Furthermore, some disasters might have been misclassified in the database.

**Fig. 2:**
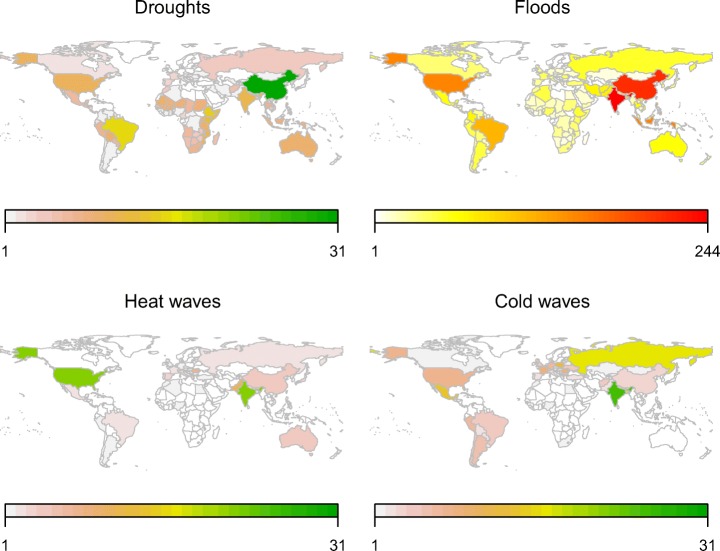
The maps show the number of climate-related disasters of each disaster type per country, which were recorded between 1950 and 2015 by EM-DAT

As climate information, we use monthly temperature and precipitation data at a 0.5^∘^× 0.5^∘^ global grid from the Climate Research Unit (CRU, Harris et al. 2014). Data in EM-DAT is in principle available from 1901 to 2015. However, we only analyze events from 1950 to 2015, since the quality and reliability of the data before 1950, for both EM-DAT and CRU, remains questionable (Harris et al. 2014). This subset contains 5006 recorded climate-related disasters. Maps indicating how many disasters of each disaster type were recorded in each country over this time period are provided in Fig. 2.

We investigate whether disasters are associated with significant climate anomalies using superposed epoch analysis (SEA) (Adams et al. 2003; Lesk et al. 2016; Nicolai-Shaw et al. 2017). For this purpose, temperature and precipitation were averaged over countries and years in which disasters were recorded. For each disaster between 1953 until 2012, we constructed a 7-year time series centering around the disaster year. Each temperature time series was normalized by subtracting the mean over the 3 years prior and 3 years following the disaster. Precipitation time series were normalized similarly by dividing by the mean of the 6 non-disaster years. We built 7-year composites for each disaster type (drought, flood, heat wave, cold wave) by averaging the 7-year time series over all disasters. Using a different number of years for compositing does not affect our results. To assess whether disasters are associated with significant climate anomalies, we constructed controls following the approach of Lesk et al. (2016). We created 1000 random disaster composites by assigning random years to disasters, keeping the number of disasters equal to the observed number. A climate anomaly is referred to as significant if the normalized composite is outside the 99th percentile of the controls (*p* < 0.01). This implies seven statistical tests for each disaster type, one test for each year. We thus adjust *p* values a posteriori for multiple testing by controlling the false discovery rate (FDR) of these seven tests following Wilks (2016).

Further analyses are based on standardized climate data. To standardize data, we linearly detrended country-averaged yearly temperature and precipitation from 1950 to 2015 and subsequently divided by their standard deviation. Controls were constructed again by randomly sampling disaster years in countries where disasters had happened.

**Fig. 3:**
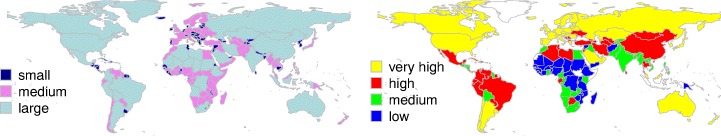
These maps show which countries belong to which country size category (left) and which countries belong to which development stage based on the Human Development Index (right). We denote all countries classified as “very high” as developed and all other countries as developing

To test the effect of country size on the magnitude of climate anomalies during disaster years, we split all countries into three country size categories: (i) small (all countries with an area < 200000*km*^2^), (ii) medium (200 000 km^2^ < country size < 1000000*km*^2^), and (iii) large (country size > 1000000*km*^2^). A map with the distribution of country size is shown in Fig. 3 (left). The categories were chosen such that each category contains a similar number of disasters.

We hypothesize that developing countries have higher vulnerability; hence, smaller climate anomalies might trigger a disaster compared to developed countries. To test for this effect, we divided countries into developed and developing countries, using the definition from the Human Development Index (HDI) (http://hdr.undp.org/en/composite/HDI), which divides all countries into one of four categories (very high, high, medium, low). This division is shown in Fig. 3 (right). We compare the set of countries in the class “very high” (referred to as “developed” in the remainder) with all countries in the three lower classes (denoted by “developing” in the remainder).

## Results

**Fig. 4:**
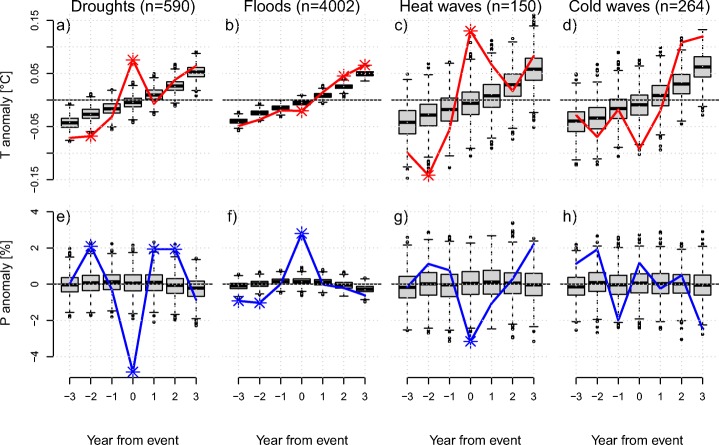
Countrywide climate anomalies from 3 years before until 3 years after a disaster. **a**–**d** Temperature anomalies in ^∘^C, **e**–**h** recipitation anomalies in % for disasters classified as droughts, floods, heat waves, and cold waves. The *n* indicates the number of events in each category. The red and blue lines show the composite event time series, centered on the event year. The box plots show 1000 control time series based on randomly sampled disaster years. Significant climate anomalies (*p* < 0.01) are marked with a star

Climate-related disasters classified as droughts, floods, and heat waves are associated with significant countrywide anomalies (*p* < 0.01) in temperature and precipitation during the year of the event (Fig. 4). Disasters classified as droughts are associated with an average increase in temperature of 0.08 ^∘^C and a decrease in precipitation of − 4.8 %. Similarly, disasters classified as heat waves are associated with an increase in temperature of 0.13 ^∘^C and a decrease in precipitation of − 3.2 %. In contrast, disasters classified as floods are associated with a decrease of temperature by − 0.02 ^∘^C and an increase in precipitation by 2.8 %. Disasters categorized as cold waves show no significant climate anomalies at the analyzed scale, although a non-significant negative deviation is evident for temperature. There is a positive trend in temperature anomalies of about 0.1 ^∘^C on average over the 7-year time period. The inter-annual variability of the disaster composite and the range of the controls are a function of the sample size and inter-annual variability of the climate in the affected countries.

**Fig. 5:**
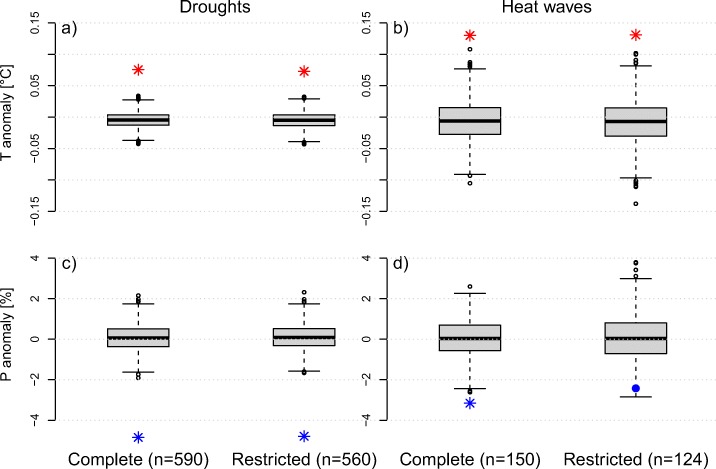
Temperature (**a**, **b**) and precipitation (**c**, **d**) anomalies and controls for drought and heat wave disasters. Shown are all disasters (complete) and the subset of disasters where droughts and heat waves in the same year were excluded (restricted). Stars mark significant anomalies (*p* < 0.01). Non-significant anomalies are presented as points

Figure 4 demonstrates that drought and heat wave disasters are associated with significant anomalies in both temperature and precipitation. To investigate potential confounding effects of multiple disasters in the same year, we remove events in years that recorded both a drought and a heat wave-related disaster. Following this procedure, we omit 26 heat waves and 30 droughts from our original data set. The exclusion of combined droughts and heat waves has little influence on temperature (Fig. 5a) and precipitation (Fig. 5c) anomalies during drought disaster years, as well as temperatures (Fig. 5b) during heat wave years. However, it does reduce the precipitation (Fig. 5d) anomaly during heat wave years below the significance threshold.

**Fig. 6:**
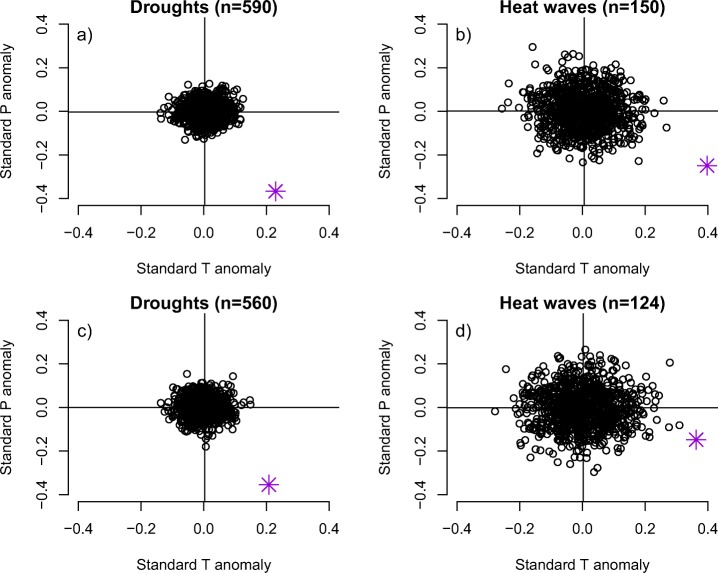
Bivariate standardized countrywide climate anomalies during drought (**a**, **c**) and heat wave disasters (**b**, **d**). Pink stars represent composites during the event years. Black circles represent 1000 controls, computed as composites with randomized event years. (**a**, **b**) All events. **(c**, **d**) Events where drought and heat wave disasters that occurred in the same year and country were removed

We investigate the potential coupling between droughts and heat waves further by conducting a bivariate analysis using detrended and standardized climate data. Composites constructed from non-disaster years show no substantial climate anomaly for either temperature or precipitation and are scattered around 0 (Fig. 6). In contrast, disaster years are associated with strong concurrent anomalies. Droughts show an average standardized precipitation anomaly of − 0.37 and an averaged standardized temperature anomaly of 0.23 (Fig. 6a). Heat waves show an averaged standardized precipitation anomaly of − 0.25 and an averaged standardized temperature anomaly of 0.39 (Fig. 6b). Excluding event years with co-occurring drought and heat wave disasters has little effect on the climate anomalies for droughts but reduces the precipitation anomaly for heat waves substantially (Fig. 6c, d). The scatter for non-disaster years is larger for heat waves due to the smaller sample size.

### The effect of country size

**Fig. 7:**
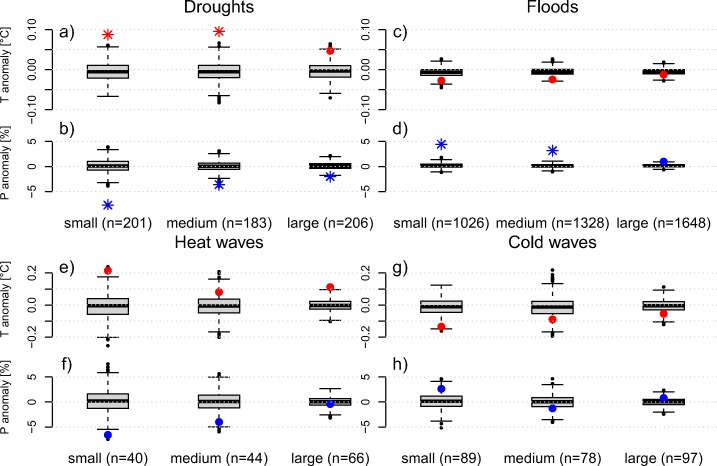
Countrywide climate anomalies during disaster years for different country sizes. Shown are temperature (red) and precipitation (blue) anomalies as annual composites for drought (**a**, **b**), flood (**c**, **d**), heat wave (**e**, **f**), and cold wave (**g**, **h**) disasters. The *n* indicates the number of events in each category. The box plots depict the 1000 controls, stars indicate significant anomalies (*α* = 0.01)

Precipitation anomalies during disasters classified as droughts, floods, and heat waves show a strong sensitivity to country size, with smaller countries experiencing larger anomalies (Fig. 7b, d, f). Temperature anomalies for these three event types show a similar behavior (Fig. 7a, c, e), albeit less strongly. Temperature and precipitation during cold waves do not show such a clear pattern (Fig. 7g, h).

**Fig. 8:**
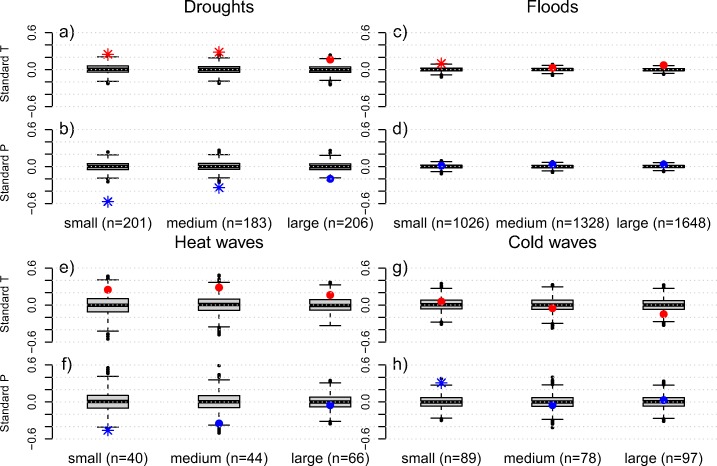
Similar to Fig. 7 but based on standardized anomalies

We plotted the same information with detrended and standardized data to investigate the confounding influence of sample size and varying inter-annual variability of the climate (Fig. 8). We find a similar decrease in the strength of the anomalies with increasing country size, most evident for precipitation during drought (Fig. 8b) and heat wave (Fig. 8f) disaster years.

### The effect of country development

**Fig. 9:**
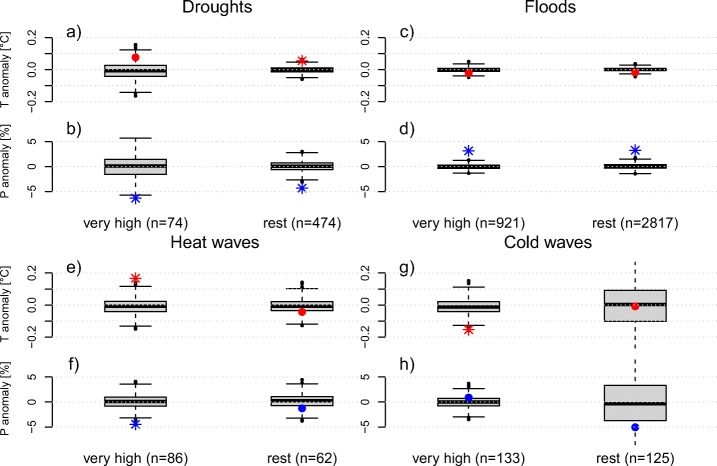
Countrywide climate anomalies during climate-related disasters for developed and developing countries. Shown are temperature (red) and precipitation (blue) anomalies as annual composites for drought (**a**, **b**), flood (**c**, **d**), heat wave (**e**, **f**), and cold wave (**g**, **h**) disasters. The *n* indicates the number of events in each category. The box plots depict the 1000 controls, stars indicate significant anomalies (*α* = 0.01)

**Fig. 10:**
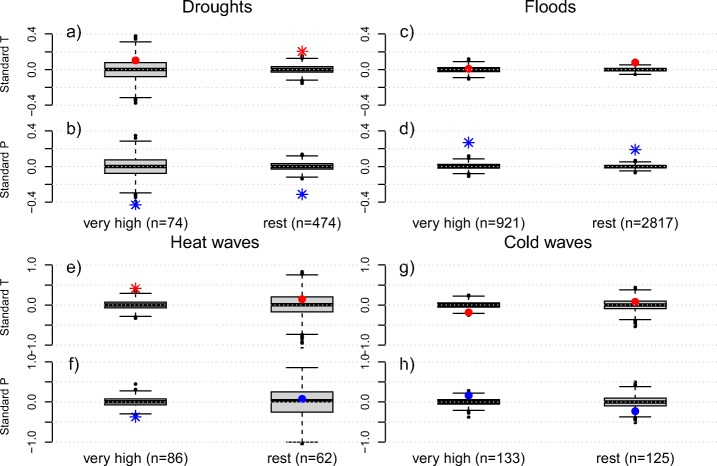
Similar to Fig. 9 but based on standardized anomalies

Whether a climate anomaly leads to a disaster largely depends on the vulnerability of a country. Temperature and precipitation during heat wave disaster years (Fig. 9e, f) as well as temperature during cold wave years (Fig. 9g) and precipitation during drought years (Fig. 9b) show stronger anomalies in developed countries as compared to developing countries. These cases support our hypothesis that developed countries need stronger climate anomalies to experience a disaster since they are less vulnerable. For disasters classified as floods (Fig. 9c, d), we could not identify this pattern. Although similar in magnitude to developing countries, the temperature anomaly in developed countries during drought years is not significant. This is likely because developed countries tend to lie in mid-latitudes, associated with higher background climate variability. Standardizing the data highlights this effect (Fig. 10). Hence, in contrast to developed countries, drought disasters in developing countries are associated with significant positive temperature anomalies in addition to a large precipitation deficit.

## Discussion

**Fig. 11:**
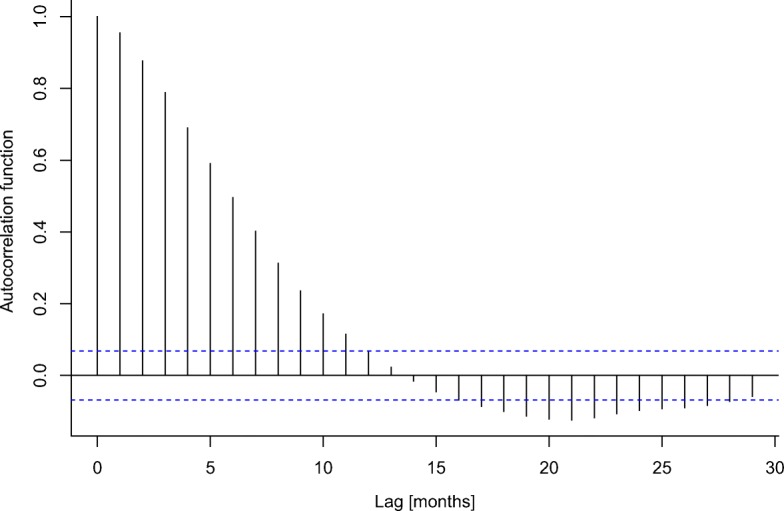
Autocorrelation of the monthly multivariate El Niño Index (MEI) from January 1950 to December 2015. Values outside the blue dashed lines are significant (*p* < 0.05)

We find highly significant anomalies in temperature and precipitation during years with disasters classified as droughts, floods, and heat waves in the EM-DAT database, even though the climate variables were averaged over entire countries and years (Fig. 4). The results demonstrate that on average, disasters are indeed linked to weather and climate events, besides the potentially large effect of vulnerability and exposure (Blaikie et al. 2004; Peduzzi 2019). The strength of the signal is surprising considering that climate information was averaged over very large spatial and temporal scales and many disasters have occurred in very large countries (Fig. 2). The positive trend in temperature composites shows the aggregated effect of climate warming. It amounts to about 1 ^∘^C over the analyzed time period 1950 to 2015. This is in line with regional warming trend estimates summarized by Hartmann et al. (2013). Occasionally, non-disaster years are associated with significant climate anomalies. These anomalies are typically much smaller than the ones in the disaster year and have the opposite sign. This is most likely an effect of the El Niño Southern Oscillation (ENSO), which leads to synchronized droughts and floods all over the globe (Ropelewski and Halpert 1987; Mason and Goddard 2001) and has significant negative autocorrelation at time lags around 20 months (Fig. 11).

While years with disasters classified as droughts and heat waves show a strong signal in both temperature and precipitation (Fig. 6), the precipitation anomaly during years with disasters classified as floods is much stronger than the temperature signal, and years with disasters classified as cold waves only show are large but non-significant anomaly in temperature during the disaster year. Hence, our analysis supports the finding that (meteorological) droughts and heat waves are strongly correlated (Mueller and Seneviratne 2012; Berg et al. 2015; Zscheischler and Seneviratne 2017). Both drought and heat wave-related disasters are associated with warmer and drier conditions (see also Fig. 6). This highlights the relevance of better understanding compound events for disaster risk management (Zscheischler et al. 2018). In particular, an intensification of the dependence between droughts and heat waves in the future (Zscheischler and Seneviratne 2017) may increase associated impacts.

The strong coupling between droughts and heat waves raises the question whether the distinction between droughts and heat waves for disasters is meaningful. Since in both cases disasters are often associated with strong anomalies in temperature and precipitation, their impacts might be similar and a distinction difficult. Removing all co-occurring drought and heat wave disasters in the same year shows little change in the climate anomalies associated with drought years and temperature anomalies during heat wave years (Fig. 5). However, precipitation anomalies during heat wave years become smaller and insignificant. This suggests that drought disasters are always accompanied by high temperatures, whereas heat wave disasters may occur without a substantial precipitation anomaly. Droughts are generally long-lived and stationary. Via soil moisture drying, they can lead to substantial increases in temperature through land atmosphere feedbacks (Seneviratne et al. 2010; Miralles et al. 2014). In contrast, the hot air masses of a heat wave may have been advected to areas with no strong precipitation deficit. This partly decoupled response for heat waves is due to the fact that, in contrast to droughts, heat waves can also only last a few days while droughts require long-term atmospheric perturbations.

Our results support the expected behavior that climate anomalies become smaller the larger the area over which they are averaged, especially for precipitation during droughts and floods (Fig. 7). Overall, precipitation shows this feature more clearly than temperature, possibly because precipitation can be very local, is less strongly correlated in space compared to temperature, and therefore flattens out more quickly when averaged over larger areas. Hence, to link disasters to climate anomalies in large countries, more detailed information on the location and timing of the disasters is required.

We find larger climate anomalies for developed countries compared to developing countries for all disasters except floods (Fig. 9). Interestingly, this separation of countries reveals a significant negative temperature anomaly for cold wave disasters in developing countries, even though the signal vanishes when the anomalies are normalized (Fig. 10). Overall, these findings support the hypothesis that a weather or climate event has to be more extreme in a developed country to result in a disaster with human impact due to smaller vulnerability (Peduzzi 2019). Developed countries tend to have more resources and a higher capacity to prevent and mitigate disaster risk, and can also better adapt after a disaster to prevent future disasters in the same region (Kreibich et al. 2017). In contrast, developing countries are already vulnerable to present-day climate extremes and may be more so in the future with further changes in climate extremes (Mirza 2003; Seneviratne et al. 2012; Bathiany et al. 2018) and adverse impacts of climate change on livelihoods (Morton 2007).

Generally, it is difficult to compare climate anomalies between the different categories in the size-dependent analysis and in the analysis of developmental level. This is because sample size and inter-annual variability, which is also related to country size, strongly affect the variability of the controls. Developing countries are mostly located in tropical and subtropical regions (Fig. 2) where inter-annual variability is much smaller than it is in temperate and polar regions. A way to control for these effects is to standardize climate data. The analysis based on standardized data confirms our findings and shows that precipitation anomalies during drought and heat wave years decrease for larger countries (Fig. 8). The results for temperature are less clear. Note that also in the standardized plots the sample size affects the variability of the controls and therefore the assessment of significance. Standardized precipitation anomalies during years with disasters classified as heat waves are indeed large and significant for developed countries, unlike for developing countries (Fig. 10). Temperature anomalies during those years follow the same pattern, being smaller and non-significant in developing countries. Precipitation anomalies during years with disasters classified as droughts are smaller in developing countries but still significant. Temperature anomalies during those years, however, show the opposite response. They are larger and significant in developing countries, in contrast to developed countries. It should be noted that the sample size for droughts in developed countries is much smaller, leading to large uncertainties. Nevertheless, these results suggest that droughts in developing countries are associated with strong positive anomalies in temperature, possibly aggravating impacts. Alternatively, these results could indicate that the information in EM-DAT is partially biased, in particular in developing countries. Note that we do not include potential changes in the developmental status over time. Some countries may have transitioned between one category to the other event though this is not well captured by the HDI. Since we only compare very highly developed countries against all others, we believe that the influence of such transitions would be very minor on our results and conclusions.

Information availability and quality on recorded disasters might be strongly limited in developing countries, leading to higher uncertainties in disaster databases (Huggel et al. 2015). For instance, data consistency tends to be smaller because institutions experience greater change and instability in developing countries. Furthermore, because of less insurance penetration in these countries, the use of disaster databases maintained by reinsurance companies is limited (Huggel et al. 2015). In general, disaster databases such as EM-DAT depend on governments, non-governmental organizations, and news agencies to provide reliable information on disaster impacts and disaster drivers. However, different agents may have different interests in what type of information becomes public. On the one hand, governments may want to keep impact numbers low or not even reveal that a disaster happened at all to show that they are in control or avoid embarrassment. On the other hand, non-governmental organizations may have an interest to report higher impact numbers to receive more donations. Taken together, these opposing interests might lead to strong uncertainties in the reported impact numbers and potentially systematic biases in the reporting of disasters. Uncertainties related to impact numbers do not affect our result, as this information has not been used in this analysis. In contrast, biases in the reporting whether a disaster has actually occurred may have an effect on our results. Nevertheless, the fact that we do find relatively strong evidence to link EM-DAT disasters to climate anomalies at the country scale, we can conclude that reporting biases are not overwhelming in EM-DAT. In that sense, our analysis provides some confidence that EM-DAT can be used for studying climate patterns related to disasters. Consequently, any improvement of the data quality should only result in even stronger relationships. To perform a more rigorous quality check, each disaster recorded in EM-DAT should be linked to a climate anomaly in the region and time when the disaster has happened, similar to case studies that have linked large impacts to preceding climate anomalies (e.g., Barriopedro et al. 2011; Hoyos et al. 2013; Cramer et al. 2014). Doing this for all 5006 disasters studied in this paper represents a major research effort and would require detailed information on the location and timing of the disaster, which is currently not publicly available. Furthermore, lagged impacts might further complicate establishing such links.

A previous study linked extreme weather disasters to crop production (Lesk et al. 2016). That analysis suggests that the likely pathway of a climatic shock to a disaster goes through reducing yields, at least for disasters classified as droughts and heat waves. Furthermore, the authors found that drought-related disasters are associated with higher yield losses in developed countries compared to developing countries. Lesk et al. (2016) suggest three possible explanations for this difference, including (i) large-scale monocultures in developed countries are more drought sensitive, (ii) smallholders in lower-income countries use risk-minimizing strategies, and (iii) generally lower fair-weather yields in lower-income countries. Our results suggest an alternative, more direct explanation for the observed pattern, which is related to a sampling bias. Developed countries can manage smaller climate shocks well, whereas similar events may lead more often to a disaster in developing countries because of higher vulnerability. This means in turn that the recorded disasters for developing countries in EM-DAT include more small- and medium-sized climate anomalies, which are consequently associated with smaller yield anomalies. In contrast, recorded disasters in developed countries are more often associated with larger climate shocks and thus larger yield anomalies.

## Conclusions

To assess future disaster risks related to climate variability, it is important to understand the climate constellations that have led to past climate-related disasters. In this study, we demonstrate that disasters recorded in the disaster database EM-DAT are associated with large climate anomalies that are still significant when averaged over complete years and entire countries. Disasters classified as droughts, floods, and heat waves are associated with significant climate anomalies during the disaster year in both temperature and precipitation. Our results suggest that developed countries require larger climate anomalies to experience a disaster, which supports the common understanding that these countries are less vulnerable to climate variability. Our study thus highlights the importance of climate hazards for disaster risk, emphasizing the relevance of well-informed climate hazard projections. To link disasters with specific climate events, more detailed information on the timing and location of disasters is necessary. In conclusion, our findings suggest that changing hazard likelihoods and intensity due to climate change will play a major role in shaping disaster risk in the future.
